# Type I Interferon Emerges as the Reproducible Core of a Shared Mitochondrial–Immune Transcriptomic Signature in Pulmonary Arterial Hypertension and Bipolar Disorder

**DOI:** 10.3390/genes17091147

**Published:** 2026-09-19

**Authors:** Cristina Ioana Stoica, Cătălin Araniciu, Ioana Andrada Ionuț, Anca Stana, Smaranda Dafina Oniga

**Affiliations:** 1 Department of Therapeutical Chemistry, Faculty of Pharmacy, UMF “Iuliu Hațieganu” Cluj Napoca, 12 Ion Creangă Street, 400012 Cluj-Napoca, Romania; stoica.cristina@umfcluj.ro (C.I.S.); araniciu.catalin@umfcluj.ro (C.A.); smaranda.oniga@umfcluj.ro (S.D.O.); 2 Department of Pharmaceutical Chemistry, Faculty of Pharmacy, UMF “Iuliu Hațieganu” Cluj Napoca, 41 Victor Babeș Street, 400012 Cluj-Napoca, Romania; stana.anca@umfcluj.ro

**Keywords:** type I interferon, peripheral blood transcriptomics, pulmonary arterial hypertension, bipolar disorder, cross-disease comparison, reproducibility, gene set enrichment analysis, weighted gene co-expression network analysis

## Abstract

**Background/Objectives:** Mitochondrial dysfunction and immune activation are reported across many chronic diseases, yet whether such shared signals are reproducible across independent cohorts is rarely tested. We compared the peripheral blood transcriptomes of two clinically distinct disorders, pulmonary arterial hypertension (PAH) and bipolar disorder (BD), to determine which shared transcriptomic signals remain directionally concordant across independent cohorts. **Methods**: Five public microarray cohorts (two PAH, three BD) were analyzed through differential expression, Hallmark gene set enrichment, and weighted gene co-expression network analysis. Instead of focusing on overlapping genes, we quantified the directional reproducibility of pathway-level signals using a stability index, evaluated against chance baselines, permutation testing, and complementary reproducibility metrics. **Results**: Both disorders converged on an overlapping mitochondrial–immune transcriptional program, and the interferon-α response was the only Hallmark pathway with positive normalized enrichment score (NES) values across all five cohorts and was significantly enriched in four of them. Beyond the interferon response, the broader pathway signature was directionally concordant across the two PAH cohorts (stability index = 0.66; Spearman ρ = 0.61) but not across the three BD cohorts (0.22; ρ = 0.14). **Conclusions**: PAH and BD share a peripheral mitochondrial–immune transcriptional program whose most reproducible element is type I interferon signaling, a signal directionally preserved across all cohorts despite differences in blood fraction. The broader transcriptomic program showed variable reproducibility that depended in part on the blood fraction sampled, highlighting the importance of evaluating both reproducibility and sample characteristics before inferring shared disease biology.

## 1. Introduction

Pulmonary hypertension is a progressive vascular disease characterized by vasoconstriction, inflammation, thrombosis, and remodeling of the pulmonary arteries, which elevate pulmonary vascular resistance and lead to right ventricular failure. In its arterial form (pulmonary arterial hypertension, PAH), it is rare but carries a poor prognosis and a rising global burden [1]. PAH shows a pronounced female predominance (female-to-male ratio approximately 2–4:1) and is most often diagnosed in the sixth decade of life, with a mean age at diagnosis of approximately 60 years. Heritable forms are driven largely by pathogenic variants in *BMPR2*, whereas other cases are associated with connective-tissue disease, congenital heart disease, and exposure to certain drugs and toxins [2]. Although its pathogenesis is multifactorial, immune and inflammatory activation, oxidative stress, and a glycolytic metabolic shift are increasingly recognized as drivers of the endothelial dysfunction and proliferation of the pulmonary artery smooth muscle that underlie this vascular remodeling [2,3,4,5]. Mitochondrial dysfunction lies at the center of this process, reprogramming cellular metabolism toward a proliferative, apoptosis-resistant vascular phenotype [5,6].

Bipolar disorder (BD) is a chronic, recurrent psychiatric illness and one of the leading causes of disability worldwide, increasingly recognized not as a purely central nervous system disorder but as a systemic condition with measurable peripheral biological correlates [7]. It affects an estimated 1–2% of the population, typically emerges in adolescence or early adulthood, follows a lifelong relapsing course, and is associated with a reduced life expectancy of roughly a decade [8]. Its etiology is strongly shaped by genetic factors, with heritability estimates of 60–85%, while environmental and stress-related factors also contribute [9]. At the molecular level, converging evidence frames BD as a disorder of neuro-immune and bioenergetic regulation, with meta-analyses reporting elevated peripheral cytokines and blood transcriptomic studies identifying interferon-stimulated gene signatures suggesting low-grade systemic inflammation and oxidative stress [10,11,12]. Bioenergetic and mitochondrial dysfunction is a recurring theme, linking impaired neuronal energy metabolism to the synaptic and mood disturbances that define the illness [13,14,15].

Despite belonging to entirely distinct clinical domains, PAH and BD converge on a set of common, non-disease-specific perturbations, including mitochondrial and bioenergetic dysfunction, oxidative stress, and chronic immune activation, all detectable in peripheral blood. In BD, these systemic alterations are well documented beyond the central nervous system, encompassing chronic low-grade inflammation, endothelial dysfunction, and substantial cardiometabolic comorbidity [7,16,17,18].

Rather than relying on clinical comorbidity, this pairing asks whether biologically convergent processes can produce shared peripheral transcriptomic signatures across two unrelated disorders. Because the same metabolic and immune programs are independently well characterized in PAH and in BD, the two provide a rigorous cross-disease framework for testing such convergence. Their clinical independence is, moreover, an analytical advantage. Because no established comorbidity or shared risk profile links the two disorders, transcriptomic signatures common to both are more plausibly attributed to genuine molecular convergence than to clinical co-occurrence. Yet mitochondrial and immune dysregulation are widespread across chronic diseases, making their mere presence in two disorders biologically unsurprising. The critical question, therefore, is not whether these signals occur in both conditions, but whether they show consistent directional concordance at the pathway level across independent cohorts, distinguishing genuine biological convergence from non-specific systemic stress. Although mitochondrial and immune dysregulation have been extensively described within disease-specific studies, whether PAH and BD share comparable peripheral transcriptomic signatures has not, to our knowledge, been systematically examined. This question is further complicated by a broader methodological problem: transcriptomic signals are often poorly reproducible across independent cohorts, with differentially expressed genes and enrichment patterns frequently failing to replicate between studies [19,20,21]. This instability is amplified in peripheral blood and in clinically heterogeneous conditions, where biological and technical variability is high. Single-disease, single-cohort designs, which predominate in the field, cannot, by construction, establish whether the direction of pathway-level changes is concordant across independent datasets. Consequently, it remains unclear whether convergent signals reflect robust biology or merely cohort-specific artifacts, making the quantification of inter-cohort stability essential to isolate genuine shared biology.

In the present study, we performed a multi-cohort cross-disease transcriptomic analysis of PAH and BD to determine whether apparently shared biological pathways are reproducible across independent cohorts. Using a uniform enrichment pipeline, we quantified directional consistency with a stability index evaluated against explicit null models and complementary reproducibility metrics. Differential expression and weighted gene co-expression network analysis were then used to examine whether pathway-level convergence was accompanied by shared diagnosis-associated genes or co-expression features in the largest cohort of each disease. Thus, rather than merely cataloging shared pathways, this study shows that cross-cohort reproducibility provides a practical criterion for identifying robust shared molecular signals and prioritizing candidates for future mechanistic and biomarker-oriented investigation.

## 2. Materials and Methods

### 2.1. Dataset Selection and Acquisition

Publicly available gene expression datasets were retrieved from the Gene Expression Omnibus (https://www.ncbi.nlm.nih.gov/geo/, accessed on 15 October 2025) using the GEOquery package [22]. We selected five human gene expression datasets: two evaluating PAH (GSE33463, GSE131793) and three evaluating BD (GSE46449, GSE39653, GSE23848). Datasets were considered eligible if they (I) profiled human peripheral blood (whole blood or a peripheral blood cell fraction), (II) followed a case–control design comparing patients with PAH or BD (without other primary psychiatric diagnoses) to controls, (III) provided retrievable data on a microarray platform with genome-wide coverage comparable to the other cohorts (RNA-sequencing datasets were excluded to ensure a uniform preprocessing and analytical pipeline across all cohorts). All datasets meeting the predefined eligibility criteria were included. Dataset selection was completed prior to downstream analyses and was independent of any observed transcriptomic concordance or biological findings. Both PAH cohorts were profiled from peripheral blood mononuclear cells (PBMC), whereas the BD cohorts comprised one whole-blood, one leukocyte, and one PBMC dataset. The potential impact of this difference was evaluated in a dedicated sensitivity analysis (Section 3.3).

Public repositories provide limited and inconsistent clinical annotation for bipolar disorder. Consequently, the included BD cohorts differed in demographic composition, tissue fraction, and available clinical characteristics (Table S8). Rather than restricting the analysis to a more homogeneous subset, we retained all eligible cohorts to evaluate whether shared transcriptomic signatures remained reproducible across independent datasets despite this expected clinical heterogeneity. Cohort characteristics are summarized in Table 1. All included datasets were generated using commercial gene expression microarray platforms from two manufacturers: Illumina, Inc. (San Diego, CA, USA), including the HumanHT-12 v3 (GPL6947), HumanHT-12 v4 (GPL10558), and Sentrix Human-6 v2 (GPL6106) BeadChips; and Affymetrix, Inc. (Santa Clara, CA, USA; now part of Thermo Fisher Scientific, Waltham, MA, USA), including the GeneChip Human Gene 1.0 ST (GPL6244) and Human Genome U133 Plus 2.0 (GPL570) arrays. The microarray platform used for each cohort is provided in Table 1.

For GSE33463, which contained several diagnostic groups, the analysis was restricted to idiopathic PAH cases versus healthy controls. Samples with interstitial lung disease, scleroderma without PAH, and scleroderma associated with PAH were excluded to avoid confounding the PAH signal with co-existing connective-tissue or pulmonary disease.

### 2.2. Preprocessing and Gene Annotation

Each dataset was preprocessed independently according to its platform. Affymetrix datasets (GSE131793, GSE46449) were processed from raw CEL files by Robust Multi-array Average normalization (affy) [28]. Among the Illumina datasets, GSE33463 was background-corrected and normalized with the neqc method (limma) [29] and filtered to probes detected (detection *p* < 0.01) in at least 50% of samples, whereas GSE39653 and GSE23848 were log2-transformed and quantile-normalized (normalizeBetweenArrays). For all datasets, probes were mapped to official gene symbols using the corresponding platform annotation and collapsed to genes by retaining, for each gene, the probe with the highest mean expression (collapseRows, MaxMean; Weighted gene co-expression analysis (WGCNA) [30]. For GSE46449, biological replicates were averaged to the subject level prior to downstream analysis.

### 2.3. Differential Expression Analysis

For each dataset, differential expression between patients and controls was assessed using limma (Table S2) [29]. A linear model was fitted with a two-group design (lmFit), the patient versus control contrast was evaluated, and statistical significance was estimated with empirical Bayes moderation (eBayes). The full ranked list of genes, ordered by the moderated *t*-statistic, was used for downstream enrichment analysis.

### 2.4. Gene Set Enrichment Analysis

Because per-gene differential expression is underpowered in small cohorts, the cross-disease comparison was conducted primarily at the pathway level using gene set enrichment analysis, which operates on the complete ranked gene list and is comparatively robust to limited sample sizes.

Pathway-level enrichment was computed for each dataset using the fgsea package (multilevel algorithm) [31] against the 50 Molecular Signatures Database (MSigDB) Hallmark gene sets [32] obtained through the msigdbr package. Genes were ranked by their limma moderated *t*-statistic, and the full ranked list of detected genes for each dataset (i.e., all genes retained after the platform-specific detection and probe-collapsing steps described in Section 2.2) was used as input to fgsea, with no significance or fold-change cutoff applied prior to enrichment. Gene identifiers were mapped between symbols and Entrez identifiers where required (clusterProfiler) [33]. Normalized enrichment scores (NES) and Benjamini–Hochberg adjusted *p*-values were retained for each Hallmark pathway in each cohort.

### 2.5. Cross-Cohort Meta-Consistency

NES values and adjusted *p*-values were assembled into a pathway-by-cohort matrix. For each Hallmark pathway, we recorded the number of cohorts in which it reached significance (adjusted *p* < 0.05) and the sign of its NES in each cohort, providing a measure of cross-cohort directional agreement that was used both to identify recurrent pathways and as the basis for the stability analysis described below.

### 2.6. Pathway Stability Index

To quantify inter-cohort reproducibility of pathway-level signals within each disease, we calculated a directional stability index, defined as the proportion of Hallmark pathways exhibiting concordant normalized enrichment score (NES) direction across all available cohorts of the same disease. Only pathways represented in at least two cohorts were evaluated. Formally, for a disease with cohorts 1 …k, the stability index is

$$Stability\;index=\left(\frac{1}{P}\right)\sum_{i=1}^{P}I\left[sign\left(NES_{i1}\right)=sign\left(NES_{i2}\right)=\cdots =sign\left(NES_{ik}\right)\right]$$

where *P* denotes the total number of pathways evaluated, and *I* is an indicator function equal to 1 when a pathway exhibited the same enrichment direction across all cohorts and 0 otherwise. The stability index therefore ranges from 0 to 1, with higher values indicating greater inter-cohort reproducibility of pathway regulation. Pathways with a normalized enrichment score of exactly zero (none in the present data) were treated as non-concordant.

We used directional (sign) concordance rather than undirected overlap because two diseases may share a pathway’s identity yet regulate it in opposite directions, which an undirected metric would not distinguish. The index was computed for PAH (two cohorts) and BD (three cohorts), both across all 50 Hallmark pathways and restricted to pathways significant in at least one cohort.

Because the probability of chance concordance depends on the number of cohorts, we derived a theoretical baseline probability. Assuming random and independent pathway rankings, the null probability of concordant direction across all cohorts is (1/2)^(*k*−1)^, where *k* is the number of cohorts. For PAH (*k* = 2), this yields a baseline of 0.50; for BD (*k* = 3), the baseline is 0.25. The stability of each disease was first compared with its own chance baseline using a one-sided exact binomial test. To compare PAH and BD directly while accounting for this difference, we used two additional approaches. First, we computed the mean pairwise NES-sign concordance across all cohort pairs within each disease. Unlike the stability index, this metric has a chance expectation of 0.50 regardless of the number of cohorts, making the two diseases directly comparable. Second, we performed a permutation test (50,000 iterations) in which NES directions were randomized independently within each cohort, generating a null distribution for the between-disease difference in stability. The stability index was insensitive to whether the full or a variance-filtered gene set was used as fgsea input, as this affected NES magnitudes but not their direction.

As complementary measures of cross-cohort reproducibility that capture enrichment magnitude rather than direction alone, we additionally computed the Spearman rank correlation of NES vectors between cohorts of the same disease, and the Jaccard index of significantly enriched pathways (both directional and undirected). Together, these metrics assess reproducibility at three levels: sign concordance (stability index and pairwise concordance), magnitude concordance (Spearman correlation), and overlap of significant pathway sets (Jaccard index).

As a robustness check, the stability index was additionally computed using the Reactome pathway collection (MSigDB C2:CP:REACTOME, 1787 gene sets).

### 2.7. Weighted Gene Co-Expression Network Analysis

Weighted gene co-expression networks were constructed for the largest cohort of each disease, GSE33463 for PAH and GSE46449 for BD, as these were the only datasets with sample sizes sufficient for stable network inference. Network analysis was performed using the WGCNA package [30] on the same samples used for differential expression analysis (idiopathic PAH versus healthy controls in GSE33463). To ensure a symmetric comparison and avoid bias introduced by disease-specific gene filtering, both networks were built from the same set of 8720 genes shared between the two microarray platforms, defined as the intersection of detected and probe-collapsed genes obtained using collapseRows (MaxMean). For GSE46449, technical replicates were averaged at the subject level prior to analysis. Sample and gene quality were assessed in both cohorts using goodSamplesGenes.

For each dataset, the soft-thresholding power was selected independently (pickSoftThreshold, powers 1–20, signed network). In GSE33463, a power of 11 met the signed scale-free topology criterion (R^2^ = 0.82). In GSE46449, no tested power reached the predefined threshold of R^2^ ≥ 0.8. The highest value observed was R^2^ = 0.79 at power 20, which was therefore retained (Figure S1).

Signed co-expression networks were constructed using blockwiseModules with a signed topological overlap matrix, a minimum module size of 30, and a merge cut height of 0.25. Module eigengenes were correlated with diagnosis using Pearson correlation, and modules significantly associated with diagnosis (*p* < 0.05) were retained for downstream analysis. Within these diagnosis-associated modules, hub genes were defined by high module membership and gene significance (|MM| > 0.7 and |GS| > 0.3).

To evaluate whether the *ANKRD46*-containing modules represented a conserved co-expression structure across diseases, their overlap was tested using a one-sided Fisher’s exact test against the shared gene universe. Functional annotation of diagnosis-associated modules was performed using Reactome, Gene Ontology (biological process), and Kyoto Encyclopedia of Genes and Genomes (KEGG) enrichment analyses through the ReactomePA and clusterProfiler packages [33,34], with Benjamini–Hochberg adjusted *p* < 0.05.

### 2.8. Software and Statistical Analysis

All analyses were performed in R 4.5.1 [35] with Bioconductor 3.21, using GEOquery 2.76.0 [22], affy 1.86.0 [28], limma 3.64.3 [29], WGCNA 1.73 [30], fgsea 1.34.2 [31], msigdbr 25.1.1, clusterProfiler 4.16.0 [33], org.Hs.eg.db 3.21.0, and ReactomePA 1.52.0 [34]. Hallmark and Reactome gene sets were obtained through msigdbr 25.1.1, which provides the MSigDB v2025.1 (Homo sapiens) release (50 Hallmark and 1787 C2:CP:REACTOME gene sets). A fixed random seed (set.seed(1)) was used for all stochastic procedures, including fgsea enrichment and the permutation test, to ensure exact reproducibility. Unless otherwise stated, statistical tests were two-sided, and *p* < 0.05 was considered significant.

## 3. Results

### 3.1. Study Design

**Figure 1:**
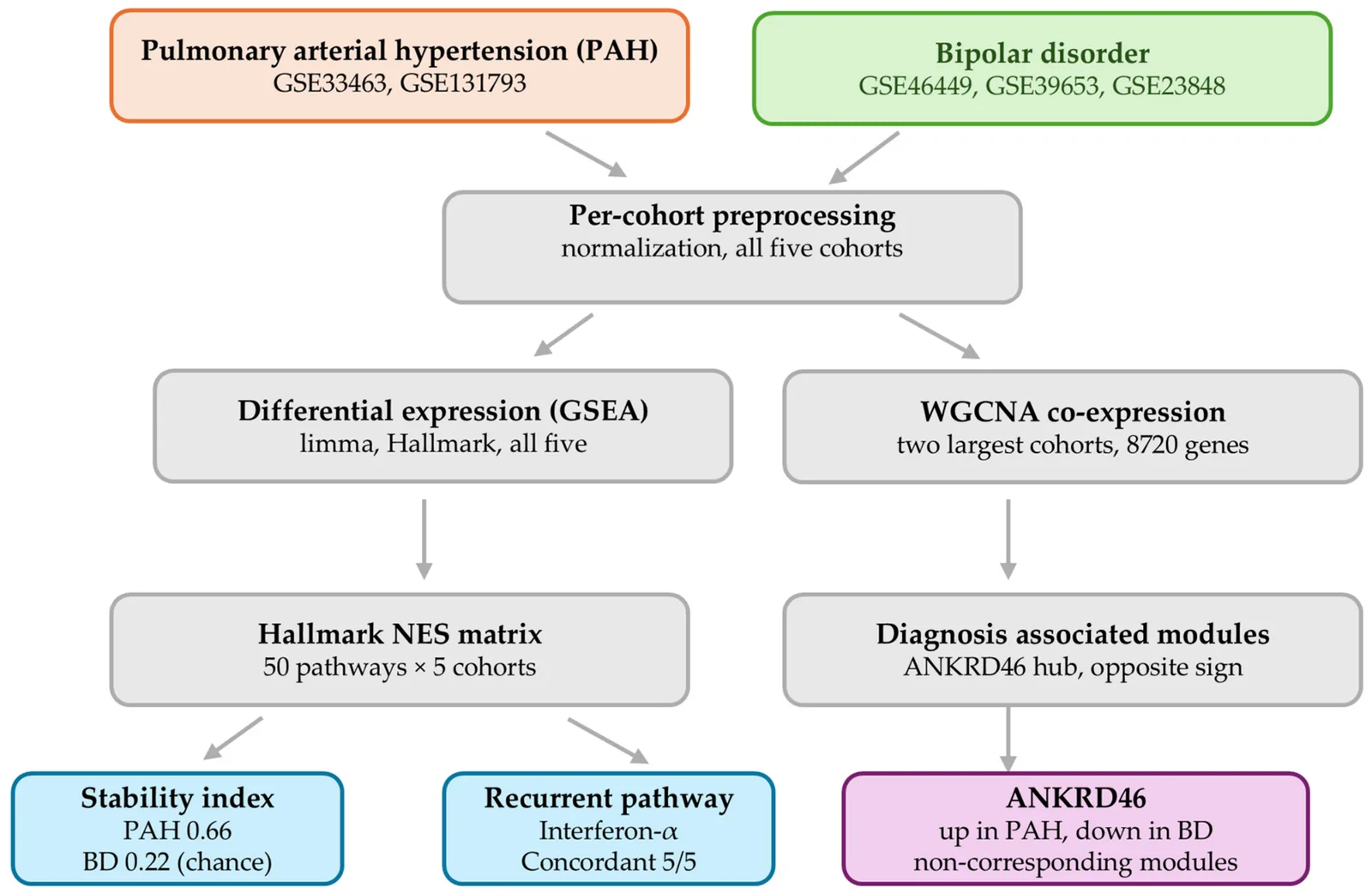
Analysis workflow.

To identify shared transcriptomic signals between PAH and BD and determine which of them are reproducible across independent cohorts, we analyzed five independent peripheral blood cohorts (two PAH, three BD; Table 1), each processed separately to preserve cohort independence (Section 2, Figure 1). Our study addressed three objectives. First, to identify biological pathways shared between PAH and BD, we determined which Hallmark pathways were recurrently enriched across cohorts. Second, to determine how consistently each disease’s pathway signature was preserved across its independent cohorts, we quantified directional concordance of enrichment using a stability index and compared PAH with BD. Third, to assess whether these shared signals also emerged at the level of gene co-expression networks, we applied weighted gene co-expression network analysis to the largest cohort of each disease, localizing diagnosis-associated modules and their hub genes. Importantly, all analyses were performed independently within each cohort before cross-cohort integration, allowing biological reproducibility to be evaluated rather than assumed.

The five cohorts differed in composition and clinical annotation (Table 1 and Table S8). Both PAH cohorts profiled PBMCs, whereas the BD cohorts were more heterogeneous, comprising one whole-blood, one leukocyte, and one PBMC dataset generated on three different microarray platforms with variable clinical annotation. Only GSE23848 reported affective state (uniformly bipolar depressed) and medication status, whereas GSE46449 included male participants only. This heterogeneity reflects the characteristics of the available public datasets and is considered explicitly in the interpretation of the analyses presented below.

### 3.2. Convergent Enrichment and Its Directional Concordance Across PAH and BD

**Figure 2:**
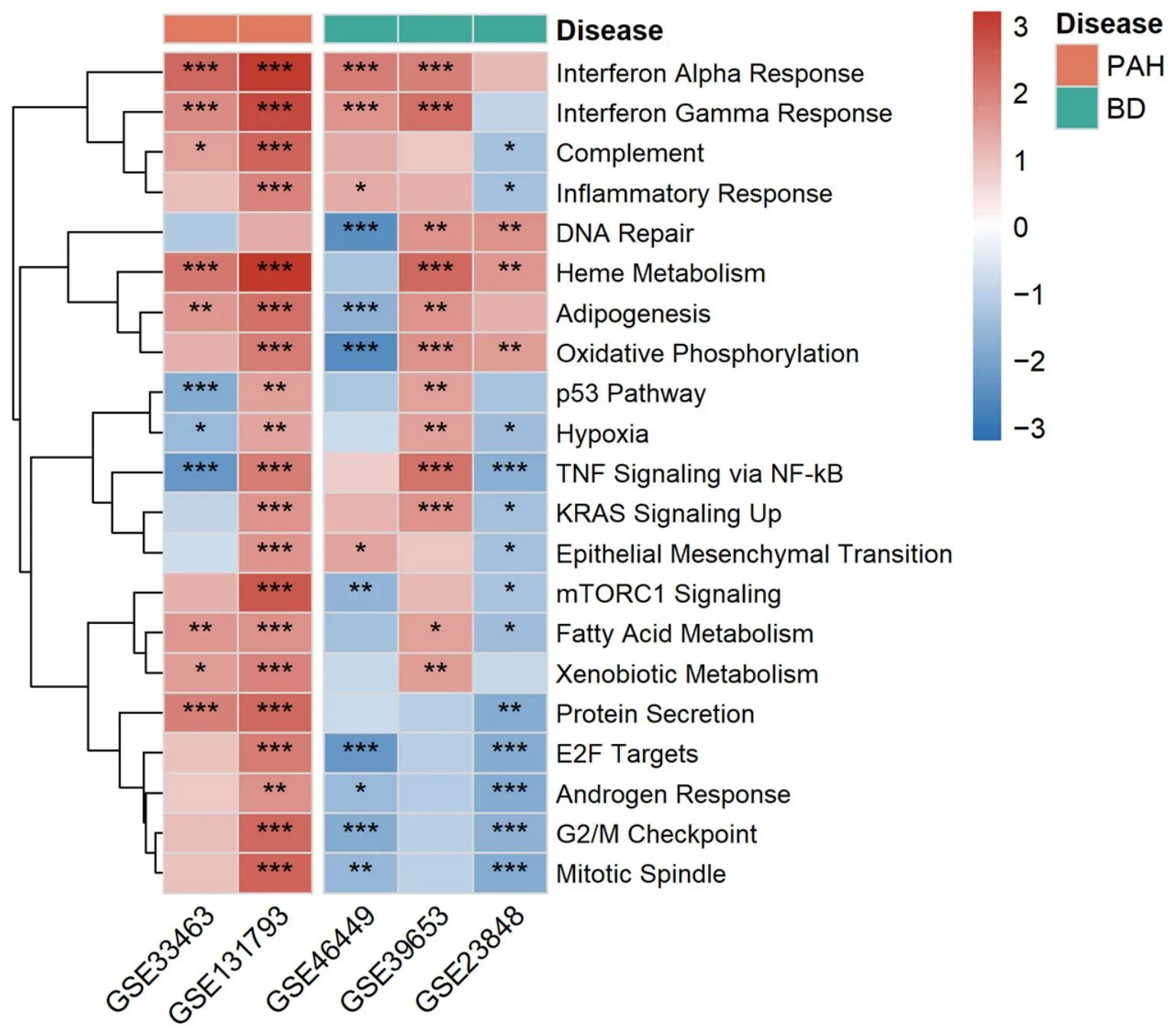
Hallmark pathway enrichment across the five cohorts. Heatmap of normalized enrichment scores (NES) for the 21 Hallmark pathways significantly enriched (adjusted *p* < 0.05) in at least three cohorts. Columns are grouped by disease (PAH: GSE33463, GSE131793; BD: GSE46449, GSE39653, GSE23848); rows are ordered by hierarchical clustering (Euclidean distance, complete linkage). Red, positive enrichment (up in patients); blue, negative enrichment. Asterisks: * adj. *p* < 0.05, ** < 0.01, *** < 0.001.

Across the five cohorts, 21 of the 50 Hallmark pathways reached significance (adjusted *p* < 0.05) in at least three cohorts and eight in at least four, but none were significant in all five (Figure 2; Table S1). While these counts capture statistical recurrence, we further examined the directionality of enrichment (NES sign) across cohorts to assess biological convergence. On this basis, the recurrent pathways resolved into two convergent biological axes: an interferon-driven immune response and mitochondrial energy metabolism, together with a minority of pathways that diverged between diseases.

The immune axis was the most consistent point of convergence. The interferon-α response (a type I interferon signature) was the only Hallmark pathway enriched in the same direction in every cohort, positive in both PAH cohorts (NES = +2.38 in GSE33463 and +3.12 in GSE131793) and in all three BD cohorts (NES = +2.09, +2.02 and +1.13), reaching significance in four of the five (adjusted *p* from 2.5 × 10^−19^ to 7.9 × 10^−5^) and remaining directionally concordant though non-significant in GSE23848. The interferon-γ response followed the same pattern, positive and significant in both PAH cohorts and in GSE46449 and GSE39653 (NES = +1.73 to +2.92) and inverted only in GSE23848 (NES = −0.93, not significant). The broader inflammatory-response and complement pathways were likewise positive in both PAH cohorts and in two of the three BD cohorts, and significantly negative only in GSE23848. Thus, across the immune axis, the two PAH cohorts agreed in direction, and the sole dissenting cohort was consistently GSE23848.

A parallel metabolic axis centered on mitochondrial energy metabolism, with a different dissenting cohort. Oxidative phosphorylation was positively enriched in both PAH cohorts, as well as in GSE39653 and GSE23848, but showed strong negative enrichment in GSE46449 (NES = −2.53, adjusted *p* = 3.5 × 10^−15^). Heme metabolism and adipogenesis displayed the same pattern, with positive enrichment in four cohorts and negative enrichment only in GSE46449. Fatty-acid metabolism was broadly similar but also showed inverse enrichment in GSE23848, making it the least consistent pathway within this group. Across the metabolic axis, the two PAH cohorts again showed concordant directionality, with GSE46449 emerging as the main source of discordance rather than GSE23848. However, not all recurrent pathways exhibited convergence. Notably, TNF signaling via NF-κB and hypoxia were enriched in opposite directions even between the two PAH cohorts, being significantly negative in GSE33463 but significantly positive in GSE131793 (TNF NES = −2.30 vs. +2.10; hypoxia NES = −1.50 vs. +1.43), and showed no consistent direction across diseases. Several proliferation- and stress-related pathways (p53, DNA repair, epithelial–mesenchymal transition) behaved similarly. These pathways illustrate that convergence between PAH and BD is selective rather than global, and that statistical recurrence does not by itself imply a shared, directionally coherent signal.

Overall, the lone dissenting cohort differed between the two convergent axes: GSE23848 for the immune pathways and GSE46449 for the metabolic pathways, while the two PAH cohorts were directionally concordant across both axes. The interferon-α response thus represented the single most reproducible point of convergence between PAH and BD, enriched in the same direction across all five independent cohorts.

Because GSE23848 diverged from the other cohorts for several immune pathways, we examined whether technical artifacts could explain this pattern. Principal-component analysis identified no outlier samples or obvious global structure (PC1 = 10.6%, PC2 = 7.5%) suggestive of a dominant technical artifact. However, subtler technical or clinical sources of heterogeneity cannot be excluded.

### 3.3. Directional Reproducibility of Pathway Enrichment Across PAH and BD Cohorts

To quantify the reproducibility of pathway-level signatures across independent cohorts within each disease, we computed a directional stability index. The index was defined as the proportion of Hallmark pathways whose NES direction was concordant across all cohorts within a given disease (Section 2.6). Because the probability of concordance by chance depends on the number of cohorts, each disease was first compared with its own baseline (0.50 for the two PAH cohorts; 0.25 for the three BD cohorts).

PAH exceeded its chance baseline. Pathway directions were concordant for 33 of 50 Hallmark pathways (stability index = 0.66), and for 30 of the 44 pathways significant in at least one cohort (0.68), both above the 0.50 expectation (one-sided exact binomial *p* = 0.016). In contrast, BD did not differ from chance. Directions were concordant across all three cohorts for only 11 of 50 pathways (0.22) and 10 of 44 (0.23), essentially equal to the 0.25 expectation (*p* = 0.74).

**Figure 3:**
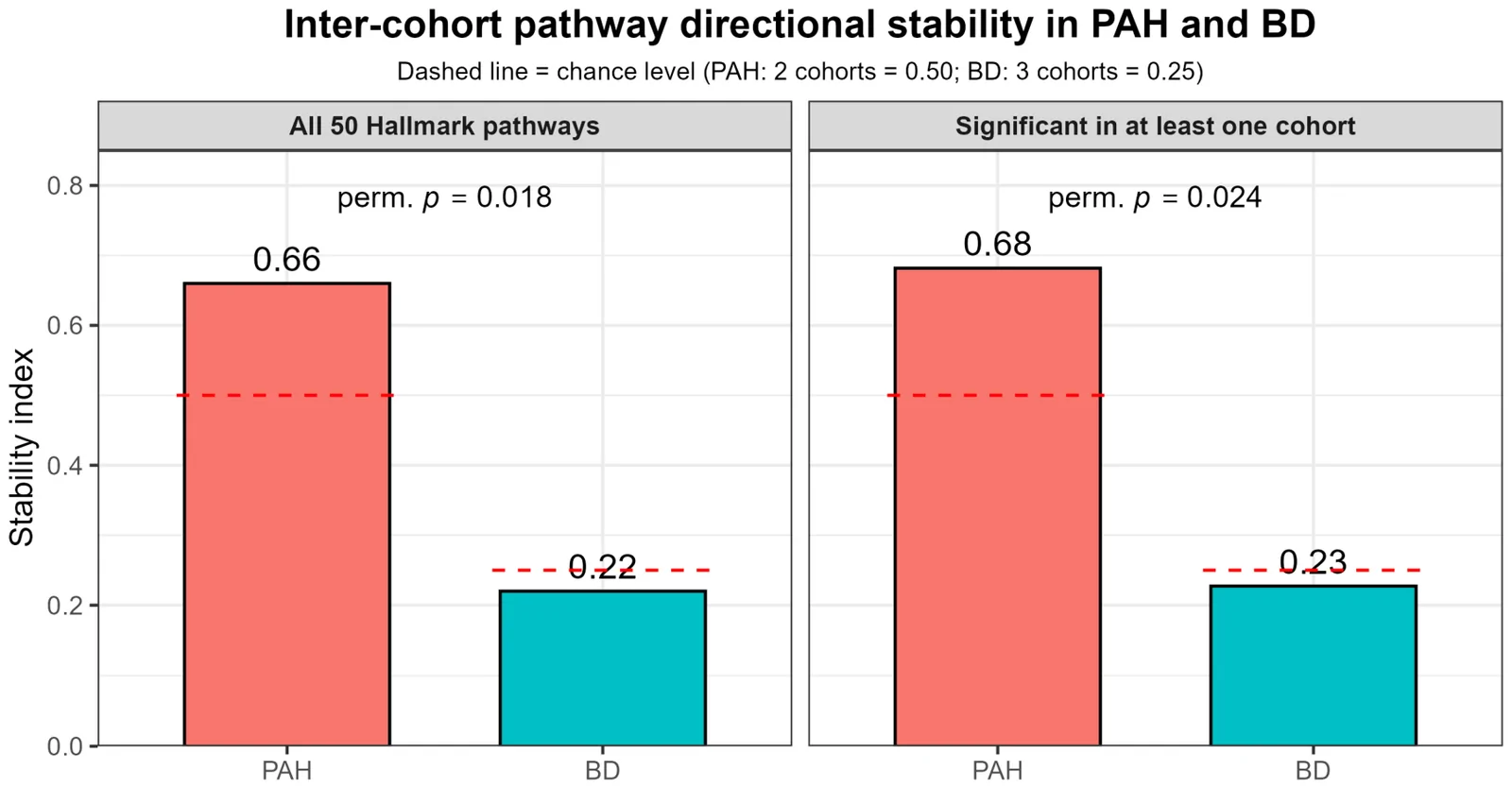
Inter-cohort directional stability of Hallmark pathway enrichment in PAH and BD. Left: all 50 Hallmark pathways; right: pathways significant (adjusted *p* < 0.05) in at least one cohort. Red dashed lines mark the chance-level baseline, which differs by the number of cohorts per disease: 0.50 for PAH (2 cohorts) and 0.25 for BD (3 cohorts).

A direct comparison confirmed this difference (Figure 3). A permutation test in which NES directions were randomized independently per cohort (50,000 iterations) showed that the between-disease difference in stability was greater than expected by chance, both across all pathways (Δ = 0.44, *p* = 0.018) and across the significant subset (Δ = 0.45, *p* = 0.024). Because the stability index depends on the number of cohorts, we also calculated the mean pairwise NES-sign concordance, for which the null expectation is 0.50 regardless of cohort count. The two PAH cohorts agreed on 66% of pathway directions, whereas mean pairwise concordance across BD cohorts was 48%, close to the 0.50 chance expectation. The between-disease difference in pairwise concordance was significant in the permutation analysis (*p* = 0.014).

Taken together, these analyses indicated that the PAH pathway signature was directionally concordant across its independent cohorts, whereas the three BD cohorts showed no such concordance across the full set. As examined in Section 4.2, cohort composition likely contributed to this difference. This contrast held even though the convergent interferon-α signal (Section 3.2) was present in both diseases, indicating that shared enrichment of a pathway does not imply that a disease’s overall transcriptomic signature is itself reproducible.

To test whether this difference depended on the binary sign-concordance metric, we benchmarked the stability index against two established measures of cross-cohort reproducibility: the Spearman rank correlation of NES vectors and the Jaccard index of significant pathways. The Spearman correlation, which uses the full magnitude of enrichment, was strong and significant for the two PAH cohorts (ρ = 0.61, *p* = 5.21 × 10^−6^) but weak for the BD cohorts (mean ρ = 0.14, individually non-significant except one pair). A direction-aware Jaccard index of significant pathways likewise favored PAH (0.22 versus a BD mean of 0.12), whereas an undirected Jaccard did not separate the two diseases (0.31 versus 0.24). Unlike the conventional Jaccard index, the direction-aware version counts a pathway as shared only when both significance and enrichment direction agree. The difference therefore lay not in the number of significant pathways, which was similar, but in their directional concordance, precisely what the stability index quantifies. To confirm that the reproducibility asymmetry was not specific to the Hallmark collection, we recomputed the stability index using the 1787 Reactome pathways (MSigDB C2:CP:REACTOME) (Table S10). The result was unchanged: PAH remained well above its chance baseline (index 0.69 over all pathways, 0.75 over the significant subset) while BD remained at chance (0.25 and 0.24). This indicates that the observed difference in directional concordance is not an artifact of the Hallmark collection.

Finally, because the three BD cohorts differed in cell fraction, we examined whether this contributed to their reduced reproducibility (Table S9). Although no single cohort consistently diverged across pathways (Section 3.2), directional concordance was higher between the two non-PBMC BD cohorts than in comparisons involving the PBMC cohort. Restricting the analysis to the two non-PBMC cohorts (GSE46449, leukocytes; and GSE23848, whole blood) increased directional concordance to 0.60 (30/50 pathways), approaching the value observed for PAH (0.66), whereas all comparisons involving the PBMC cohort (GSE39653) remained at chance level (0.42). Thus, the PBMC cohort differed broadly from both non-PBMC cohorts across many pathways, rather than only within specific biological processes. This pattern is consistent with blood fraction contributing to the reduced concordance, although blood fraction could not be disentangled from other cohort-specific differences because only one BD PBMC cohort was available.

### 3.4. A Shared but Oppositely Directed Hub Gene (ANKRD46) in Non-Corresponding Modules

**Figure 4:**
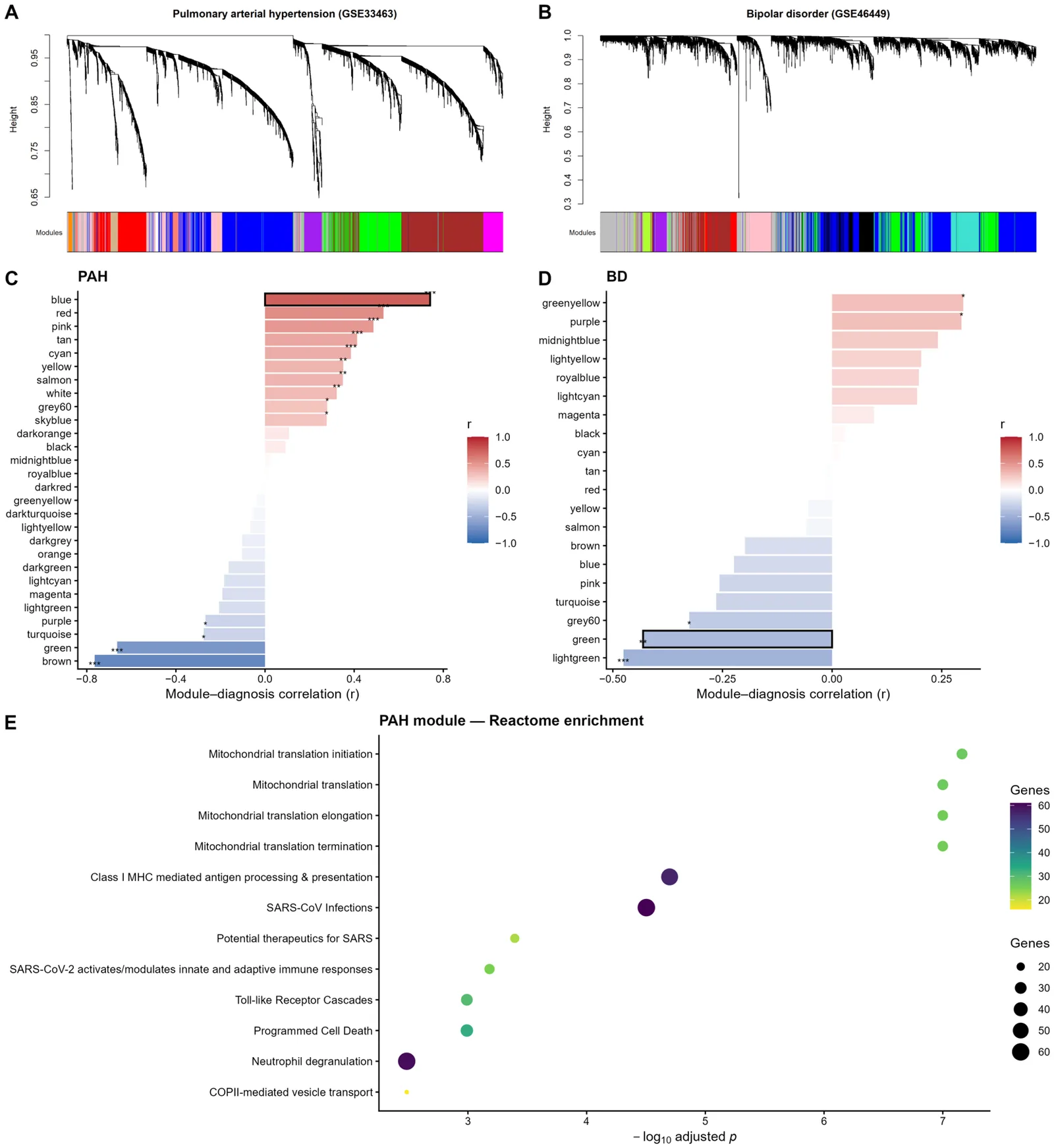
Weighted gene co-expression network analysis (WGCNA) of PAH (GSE33463) and BD (GSE46449). Both networks were built from the 8720 genes common to the two platforms. (**A**,**B**) Gene clustering dendrograms with module assignments (color bar) for PAH (**A**) and BD (**B**). (**C**,**D**) Module eigengene–diagnosis correlation for PAH (**C**) and BD (**D**); bars colored by Pearson r (red, positive; blue, negative), the *ANKRD46*-containing module outlined in black, asterisks denoting significance (* *p* < 0.05, ** *p* < 0.01, *** *p* < 0.001; gray module omitted). (**E**) Reactome enrichment of the PAH *ANKRD46*-containing module, ranked by −log_10_ adjusted *p* and sized by gene count; infectious-disease labels reflect shared interferon-stimulated genes, not active infection.

To determine whether the shared signal re-emerged at the level of gene co-expression, we constructed WGCNA networks for the largest cohort of each disease using a common set of 8720 genes (Section 2.7). In PAH, the analysis identified 29 modules, of which the module containing *ANKRD46* showed the strongest positive correlation with IPAH status (*r* = +0.74, *p* = 1.5 × 10^−13^; Figure 4A,C). In BD, the network resolved into 21 modules, and the module containing *ANKRD46* was inversely associated with diagnosis (*r* = −0.43, *p* = 1.1 × 10^−3^; Figure 4B,D).

*ANKRD46* emerged as a hub gene within diagnosis-associated modules in both diseases, but with opposite directional relationships (Table S3). In PAH, it occupied a central position in the positively associated module (|MM| = 0.74, |GS| = +0.50), consistent with its significant up-regulation in the same cohort by differential expression analysis. In BD, it was similarly central within the inversely associated module (|MM| = 0.88, |GS| = −0.47) and was down-regulated in patients.

Notably, the two *ANKRD46*-containing modules were not themselves conserved across diseases. They shared no more genes than expected by chance (51 observed versus 67.6 expected; Fisher’s exact *p* = 0.99, odds ratio = 0.71), indicating that *ANKRD46* occupied distinct co-expression contexts in each network. Thus, its recurrence represents a context-dependent gene-level finding, characterized by network centrality and opposite expression direction, rather than preservation of a shared co-expression module.

To assess whether this opposite regulation extended beyond the two cohorts used for network analysis, we examined *ANKRD46* across all five datasets (Table S7). It was significantly up-regulated in the larger PAH cohort (GSE33463, log_2_ fold-change = +0.60, adjusted *p* = 3.4 × 10^−5^) and down-regulated in the two larger BD cohorts (GSE46449 and GSE23848: log_2_ fold-change = −0.27 and −0.36, respectively), whereas its log_2_ fold-change was near zero in the two smallest cohorts (GSE131793, −0.10; GSE39653, +0.04). The opposite pattern was most evident in the larger cohorts but was not consistently observed across all five datasets.

The two modules also differed markedly in detectable functional enrichment: the PAH module returned numerous enriched terms, whereas the BD module returned almost none. This difference persisted when both networks were built from the same gene set, indicating that it was not attributable to asymmetric gene filtering. The PAH module was functionally rich, yielding 297 Gene Ontology (biological process) terms, 33 KEGG pathways, and 67 Reactome pathways (Tables S4–S6). These recapitulated the mitochondrial–immune axis already seen by differential expression and Hallmark enrichment, with leading terms including mitochondrial gene expression and translation, defense/response to virus (reflecting interferon-stimulated genes rather than active infection), and Golgi/vesicle transport (Figure 4E and Figure S2). In contrast, the BD module yielded no significant Gene Ontology terms and only one KEGG and one Reactome pathway despite containing 507 annotated genes. The BD module yielded substantially less functional enrichment under the applied annotation frameworks than the PAH module. However, these observations originate from different analyses, one within a single cohort and the other across cohorts, and therefore should not be assumed to reflect the same underlying mechanism.

This functional asymmetry was not specific to the *ANKRD46*-containing modules: the most strongly diagnosis-associated BD module comprised only 42 genes and returned no enriched terms, whereas the corresponding PAH modules were large and functionally rich.

## 4. Discussion

This study compared the peripheral blood transcriptomes of PAH and BD to examine whether the two disorders share transcriptomic alterations and, more importantly, how reproducibly those alterations replicate across independent cohorts of each disease. Using a uniform Hallmark enrichment pipeline across five cohorts, our analysis yielded three principal findings. First, both disorders engaged an overlapping mitochondrial–immune program. Second, this program was directionally reproducible across the two PAH cohorts, whereas reduced reproducibility across the three BD cohorts was partly associated with differences in cohort composition. Third, a single gene, *ANKRD46*, recurred as a hub of a diagnosis-associated co-expression module in both diseases, yet in opposite directions. Together, these observations reframe a conventional cross-disease comparison around the question of reproducibility and the factors that shape it.

### 4.1. A Shared but Non-Specific Mitochondrial–Immune Axis

The recurrently enriched pathways resolved into two coherent biological groups: a type I interferon-driven immune response and mitochondrial energy metabolism. The interferon-α response was the only Hallmark pathway directionally concordant across all five cohorts, reaching statistical significance in four. Both components are biologically plausible in each disease. Type I interferon and antiviral signaling have been linked to pulmonary vascular inflammation and remodeling in PAH [36,37,38] and reported as interferon-stimulated gene signatures in the blood of patients with BD [39,40]. Likewise, mitochondrial dysfunction and altered energy metabolism have long been recognized as central features of pulmonary vascular remodeling [6] and bipolar disorder [13,15]. The recurrence of this mitochondrial–immune axis across Hallmark enrichment and the functional annotation of the PAH co-expression module provides internal consistency across complementary analytical levels. Notably, this mitochondrial–immune annotation emerged from the PAH co-expression module even when both networks were built from an identical gene set, whereas the corresponding BD module yielded limited significant functional enrichment, indicating that the difference was not solely attributable to asymmetric gene filtering.

However, mitochondrial and immune dysregulation are not specific to PAH or BD, being broadly implicated across a range of chronic somatic and neuropsychiatric diseases [41,42]. This analysis contributes by quantifying the directional reproducibility of that shared axis rather than its mere presence. The type I interferon signature was directionally preserved across all five cohorts, whereas oxidative phosphorylation varied across the BD cohorts. *ANKRD46* represented a distinct form of divergence, recurring at the network level but showing opposite regulation in PAH and BD.

More specifically, the shared type I interferon response may reflect a broadly conserved response to cellular stress or disrupted homeostasis rather than a disease-specific mechanism. Type I interferon signaling is a pleiotropic response that can be activated across diverse pathological conditions and is associated with cellular stress, inflammation, and immune dysregulation [43,44]. Its recurrence across these biologically distinct disorders may therefore be more consistent with a common systemic response to homeostatic disturbance than with a shared disease-specific pathway.

A plausible mechanistic link may connect the two axes identified here. Mitochondrial stress and dysfunction can promote the release of mitochondrial DNA into the cytosol, where it can be sensed by the cyclic GMP–AMP synthase–stimulator of interferon genes (cGAS–STING) pathway and induce a type I interferon response through IRF3 activation [45]. This nucleic-acid-sensing mechanism operates across diverse pathological states, including inflammatory, metabolic, and autoimmune conditions, and can be engaged by cellular stress in addition to infection [46]. Conversely, immune-cell activation can induce metabolic reprogramming that perturbs mitochondrial homeostasis. The co-occurrence of mitochondrial and type I interferon signatures in peripheral blood may therefore reflect a single interconnected process, in which mitochondrial perturbation contributes to interferon activation through innate immune sensing, rather than two entirely independent axes. This interpretation is consistent with the possibility that the interferon signature represents a broadly deployed response to disrupted cellular homeostasis, as discussed above.

### 4.2. The Reproducibility of the Shared Signature Varies with Cohort Composition

A pathway can be enriched in two diseases while the broader transcriptomic signature is far less reproducible across the independent cohorts of one of them. Distinguishing a genuinely reproducible signal from an apparently shared one therefore requires testing directional concordance across independent cohorts, not merely cataloging overlapping pathways.

Across the two available PAH cohorts, the pathway signature showed significant directional concordance, whereas for the three available BD cohorts, concordance did not exceed the corresponding chance expectation. This contrast was consistent across the reproducibility metrics applied, including the directional stability index, pairwise concordance, permutation testing, and the Spearman correlation of NES vectors (ρ = 0.61 for PAH versus a mean of 0.14 for BD). An undirected overlap of significant pathways did not distinguish the two diseases, demonstrating that reproducibility depended on the direction rather than the number of enriched pathways. This apparent contrast, however, should not be attributed to disease biology alone, as cohort composition provided an additional and potentially important source of variation. This interpretation is consistent with previous evidence that peripheral blood transcriptomic studies of BD are often underpowered and yield heterogeneous signatures [47]. These signatures may also be influenced by medication, particularly lithium, which has substantial effects on the blood transcriptome [48], as well as by affective state [49,50].

Earlier studies described the poor cross-study consistency of BD blood signatures qualitatively. Here, the stability index provides an explicit measure of pathway-direction concordance and evaluates it against a defined null expectation. Framed this way, the variability in cross-cohort reproducibility is itself an informative result: it cautions against treating single-cohort BD enrichment signatures as established biology. More importantly, it illustrates that reproducibility itself provides information beyond pathway identity and should therefore be considered when comparing disease transcriptomes.

The directional concordance observed across the BD cohorts was not uniform. In an exploratory analysis, reproducibility between the two non-PBMC cohorts approached that observed for PAH, whereas concordance remained at chance level in comparisons involving the PBMC cohort (Section 3.3). This pattern is consistent with differences in cell composition.

The type I interferon response provides a useful illustration because it represents a broadly distributed transcriptional program. Interferon-stimulated genes are expressed across multiple circulating immune cell populations and are therefore expected to be detected in PBMC, whole-blood, and leukocyte samples, although granulocytes present in whole blood and leukocyte preparations may further amplify the response [51]. This broad cellular distribution may help explain why the interferon-α response remained directionally concordant across all five cohorts. By contrast, pathways dominated by cell populations excluded from PBMC, particularly granulocytes, may be less consistent across sample types. Because only one BD PBMC cohort was available, however, the contribution of blood fraction cannot be separated from other cohort-specific differences.

The increased concordance between the two non-PBMC BD cohorts is therefore consistent with an effect of cellular composition but does not establish it as the primary cause of BD heterogeneity. More broadly, the analysis shows that cohort composition can materially influence estimates of transcriptomic reproducibility and should be considered before shared pathway signals are assigned a disease-specific biological interpretation.

To further assess whether cell-type distribution could contribute to the observed patterns, we examined the expression of *ANKRD46* and interferon-stimulated genes across circulating immune cell populations using publicly available single-cell reference data [52]. *ANKRD46* showed relatively low cell-type specificity and was expressed across granulocytes, monocytes, T cells, B cells, NK cells, and dendritic cells, without pronounced enrichment in any single lineage [53]. Its broad cellular distribution makes a simple explanation based on differences in the abundance of a single immune-cell population less plausible and is more compatible with context-dependent transcriptional regulation, as discussed above. By contrast, interferon-stimulated genes such as *ISG15* were also expressed across multiple circulating immune-cell populations rather than being restricted to a single lineage. This broad cellular distribution provides a plausible cell-level explanation for the reproducibility of the interferon signature across blood-derived datasets. Because interferon-stimulated genes can be induced across diverse circulating immune-cell populations, their coordinated expression may remain detectable across blood-derived datasets despite differences in sample composition, including whole blood, leukocyte-enriched samples, and peripheral blood mononuclear cells.

The influence of treatment exposure on these signals is therefore directly relevant. Peripheral blood transcriptomic differences in BD have been reported to be driven, at least in part, by lithium exposure rather than by diagnosis itself [49]. Moreover, lithium has been shown to directly modulate immune cell populations, increasing the proportions of CD14+ monocytes and dendritic cells in patient-derived peripheral blood mononuclear cells [54], both of which are relevant to type I interferon biology. These findings support the interpretation that medication exposure may contribute substantially to the between-cohort variability observed in BD and that both *ANKRD46* expression and interferon-related signals should be considered in the context of treatment status. Such effects could contribute to cohort-dependent variation, whereas the recurrent interferon signature observed across cohorts with differing treatment profiles appears comparatively more robust to these differences.

### 4.3. ANKRD46 as a Recurrent but Oppositely Directed Node

*ANKRD46* was up-regulated in PAH and belonged to a module positively associated with diagnosis, whereas in BD it was down-regulated and belonged to a negatively associated module. Critically, this recurrence did not reflect a conserved module. When both networks were constructed from an identical set of common genes, the *ANKRD46*-containing modules of the two diseases shared no more genes than expected by chance (Fisher *p* = 0.99), and *ANKRD46* occupied a different module in each. The recurrence therefore identifies *ANKRD46* as a context-dependent candidate rather than evidence of a conserved co-expression program. Because each module was large and contained many genes meeting the hub thresholds, *ANKRD46* is best regarded as one of several hubs rather than a unique driver. Nonetheless, it was the only gene that was simultaneously a network hub in both diseases and differentially expressed while being regulated in opposite directions, marking it as a shared but divergently regulated node rather than a uniformly altered marker. *ANKRD46* encodes a poorly characterized ankyrin repeat domain protein with no well-defined physiological function (UniProt Q86W74) and no robust prior genetic association with either disorder. This limited biological annotation warrants cautious interpretation while supporting further investigation of *ANKRD46* as a candidate regulatory node.

Consistent with this interpretation, *ANKRD46* has recently been identified in a comparable network-based analysis as a shared, experimentally validated diagnostic marker in another biologically unrelated disease pair, keloid and type 2 diabetes mellitus, where it was up-regulated in both conditions and associated with immune-cell populations, including plasmacytoid dendritic cells, as well as with IL6–JAK–STAT3 signaling [55]. Its recurrence as a co-expression hub across multiple, biologically distinct disease contexts supports the view that *ANKRD46* may represent a biologically relevant node warranting dedicated functional investigation. Of particular interest, its reported association with plasmacytoid dendritic cells, a major cellular source of type I interferon, provides a potential link to the interferon signature identified in the present study and raises the possibility that *ANKRD46* may participate, directly or indirectly, in interferon-related immune regulation. This hypothesis remains to be tested experimentally. Importantly, the direction of *ANKRD46* regulation is not consistent across disease contexts, being up-regulated in keloid and type 2 diabetes but showing opposite directions of association between PAH and BD in our datasets. This context dependence suggests that its biological role may be shaped by disease-specific cellular and regulatory environments rather than reflecting a uniform transcriptional response.

### 4.4. Blood Versus Brain and Tissue of Origin

A recurring question for blood-based transcriptomics in a brain disorder is how faithfully peripheral signal reflects central pathology. Increasing evidence indicates that peripheral blood captures systemic immune and metabolic dimensions of neuropsychiatric disease that overlap with, but do not fully recapitulate, tissue-specific processes. Consistent transcriptomic alterations have been described across multiple neurodegenerative and psychiatric disorders in the brain [56] and, although less consistently, in peripheral blood [57], with dysregulated type I interferon signaling emerging as a recurrent feature [58]. For PAH, the relevant pathology resides in the pulmonary vasculature and right heart, and for BD in the central nervous system. In neither case does peripheral blood provide direct access to the affected tissue. The shared interferon and mitochondrial signals identified here are therefore best interpreted as systemic correlates detectable in a clinically accessible compartment rather than direct readouts of pulmonary vascular or neuronal pathology. Rather than serving as a surrogate for the primary affected tissue, peripheral blood may therefore provide a window into systemic biological processes that are shared across otherwise distinct diseases.

### 4.5. Limitations

Several limitations should be considered when interpreting these findings. First, the analysis relied on publicly available datasets with incomplete clinical metadata, precluding systematic adjustment for medication and other clinical covariates. Patients with BD are commonly treated with lithium, mood stabilizers, and antipsychotics, all of which influence the peripheral blood transcriptome [48], while patients with PAH receive vasodilators and anticoagulants and experience chronic hypoxia. Consequently, disease- and treatment-related effects cannot be fully disentangled. Second, transcriptomic associations in blood are correlative and cannot distinguish primary disease drivers from secondary systemic responses. Our findings identify reproducible correlates, not causes. Third, the BD cohorts were not harmonized for affective state (mania, depression, or euthymia), which is known to modulate the blood transcriptome [50] and which plausibly contributes to the low cross-cohort reproducibility we observed. Fourth, peripheral blood only partially mirrors the affected tissue in either disease and does not capture signals specific to the central nervous system or the pulmonary vasculature [57]. Fifth, the cohorts were modest in size, most notably GSE131793 (n = 20), which yielded no individually significant differentially expressed genes, and were generated on different microarray platforms, so quantitative effect sizes should be interpreted with caution even though our pathway- and direction-level analyses are comparatively robust to these differences. Despite the absence of significant individual differentially expressed genes, GSE131793 contributed numerous significant pathway enrichments, supporting its inclusion in the pathway-level analyses. Sixth, several enriched immune terms are annotated by Reactome and KEGG under infectious-disease labels (e.g., SARS-CoV-2, HIV, hepatitis C virus, *Salmonella enteritidis*, measles virus (gen *Morbillivirus*). These reflect shared interferon-stimulated and antiviral genes rather than active infection and should not be read as evidence of an infectious etiology. Finally, all analyses are computational and hypothesis-generating. Experimental validation (for example by quantitative reverse transcription PCR or functional assays in disease-relevant models) will be required to confirm the biological relevance of the shared axis and of *ANKRD46* in particular. In addition, the stability index quantifies directional reproducibility but does not capture differences in enrichment magnitude and should therefore be interpreted as complementary to established measures of transcriptomic similarity rather than as a replacement for them.

The BD cohorts also differed in affective state, medication exposure, sex composition, blood fraction, and microarray platform (Table S8), all of which may have contributed to reduced cross-cohort concordance. Notably, two of the cohorts were sex-homogeneous in opposite directions (GSE131793, female only; GSE46449, male only), which may further contribute to their divergent behavior. In particular, concordance increased when the analysis was restricted to the two non-PBMC cohorts. However, because only one BD PBMC cohort was available, blood fraction was confounded with cohort identity and its independent contribution could not be estimated. The sensitivity analysis should therefore be regarded as exploratory evidence that sample type affects reproducibility, rather than as proof that blood fraction was the primary source of heterogeneity.

## 5. Conclusions

Pulmonary arterial hypertension and bipolar disorder share a peripheral-blood mitochondrial–immune transcriptomic program whose most reproducible component was a type I interferon signature, directionally concordant across all five cohorts and statistically significant in four. This interferon core was the most robust shared molecular signal identified, showing that a systemic transcriptional program can remain detectable across clinically unrelated disorders and heterogeneous cohorts. In contrast, reproducibility of the broader program differed between the available PAH and BD cohorts and was partly associated with cohort composition. Restricting the BD analysis to the two non-PBMC cohorts increased directional concordance, suggesting that sample characteristics contribute to the apparent asymmetry but do not fully explain it. *ANKRD46* emerged as a recurrent yet oppositely regulated network hub, supporting its prioritization for future functional investigation rather than its interpretation as an established mechanistic driver. Collectively, these findings show that cross-cohort directional reproducibility and cohort composition should be evaluated before shared peripheral-blood transcriptomic signatures are interpreted as evidence of common disease mechanisms.

## Figures and Tables

**Table 1:** Transcriptomic datasets included in the cross-disease analysis.

Dataset	Condition	Platform (GPL)	Tissue	Patients	Controls	Total	Sex (F/M)
GSE33463 [23]	PAH	Illumina HumanHT-12 V3 (GPL6947)	PBMC	30	41	71	Not reported
GSE131793 [24]	PAH	Affymetrix Human Gene 1.0 ST (GPL6244)	PBMC	10	10	20	20/0
GSE46449 [25]	BD	Affymetrix HG-U133 Plus 2.0 (GPL570)	Leukocytes (PBL)	29	25	54	0/54
GSE39653 [26]	BD	Illumina HumanHT-12 V4 (GPL10558)	PBMC	8	24	32	Not reported
GSE23848 [27]	BD	Illumina Sentrix Human-6 v2 Expression BeadChip (GPL6106)	Whole blood	20	15	35	24/11

*PAH, pulmonary arterial hypertension; BD, bipolar disorder; PBMC, peripheral blood mononuclear cells; PBL, peripheral blood leukocytes; GPL, GEO platform accession.*

## Data Availability

All datasets analyzed in this study are publicly available from the Gene Expression Omnibus (https://www.ncbi.nlm.nih.gov/geo/ accessed on 25 October 2025) under accession numbers GSE33463, GSE131793, GSE46449, GSE39653, and GSE23848. The complete analysis code is openly available at Zenodo (DOI: 10.5281/zenodo.21290266). All processed results supporting the findings are provided in the Supplementary Materials.

## Supplementary Materials

The following supporting information can be downloaded at: https://www.mdpi.com/article/10.3390/genes17091147/s1. Figure S1: Soft-thresholding power selection for the WGCNA networks; Figure S2: Functional enrichment of the PAH co-expression module; Table S1: Hallmark GSEA (NES and adjusted *p*) for all 50 pathways across the five cohorts; Table S2: Differential expression results (limma) for each cohort; Table S3: WGCNA hub genes of the diagnosis-associated modules; Table S4: Reactome enrichment of the co-expression modules; Table S5: Gene Ontology (biological process) enrichment of the PAH module; Table S6: KEGG pathway enrichment of the co-expression modules; Table S7: *ANKRD46* differential expression (log2FC, *p*) across all five cohorts; Table S8: Available clinical and demographic metadata for the three BD cohorts; Table S9: Tissue-stratified sensitivity analysis of the BD stability index (whole blood vs. PBMC); Table S10: Robustness of the stability index to gene-set collection (Hallmark versus Reactome).

## Abbreviations

The following abbreviations are used in this manuscript:

PAH	Pulmonary arterial hypertension
BD	Bipolar disorder
PBMC	Peripheral blood mononuclear cells
WGCNA	Weighted gene co-expression network analysis
NES	Normalized enrichment score
MM	Module membership
GS	Gene significance
PBL	Peripheral Blood Leukocytes
KEGG	Kyoto Encyclopedia of Genes and Genomes
MSigDB	Molecular signatures database
