# Supplementary material for: Type I Interferon Emerges as the Reproducible Core of a Shared Mitochondrial–Immune Transcriptomic Signature in Pulmonary Arterial Hypertension and Bipolar Disorder
Source: Genes (Basel). 2026 Sep 19;17(9):1147. doi: 10.3390/genes17091147 (PMC13606078; doi:10.3390/genes17091147)
Supplement: Supplementary file 1 [file genes-17-01147-s001.zip › Supplementary_Figures.pdf]

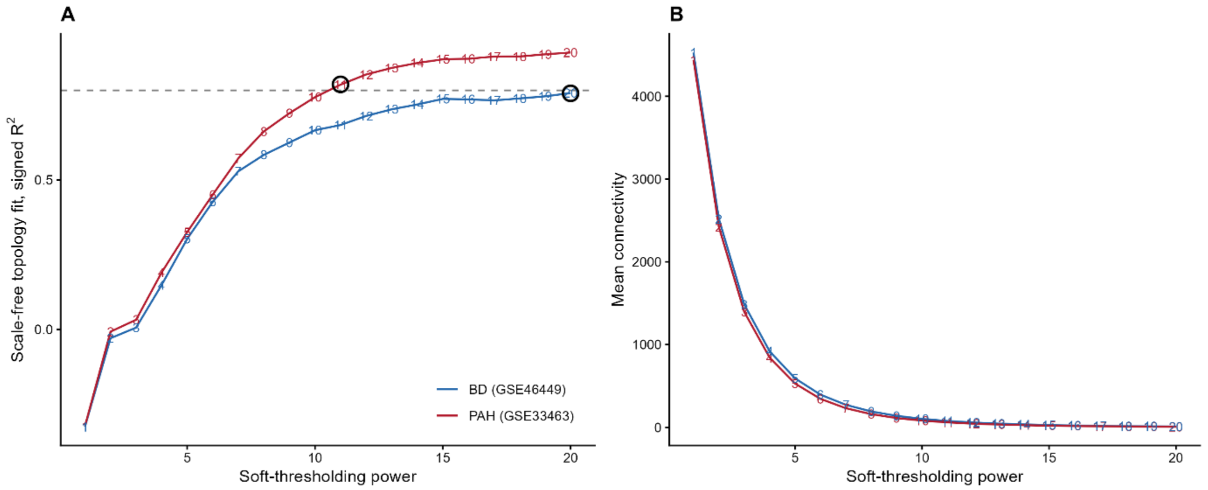

Figure S1. Soft-thresholding power selection for the symmetric WGCNA networks. Signed scale-free topology fit (R^2) and mean connectivity as a function of soft-thresholding power (1–20) for the PAH (GSE33463) and BD (GSE46449) cohorts, both constructed from the same 8,720 genes common to the two platforms. The dashed line marks the $R^2 = 0.8$ criterion. A power of 11 satisfied this criterion in GSE33463 ($R^2 = 0.82$), whereas GSE46449 did not reach $R^2 \geq 0.8$ at any tested power (maximum $R^2 = 0.79$ at power 20, which was retained), consistent with its weaker, more diffuse co-expression structure.

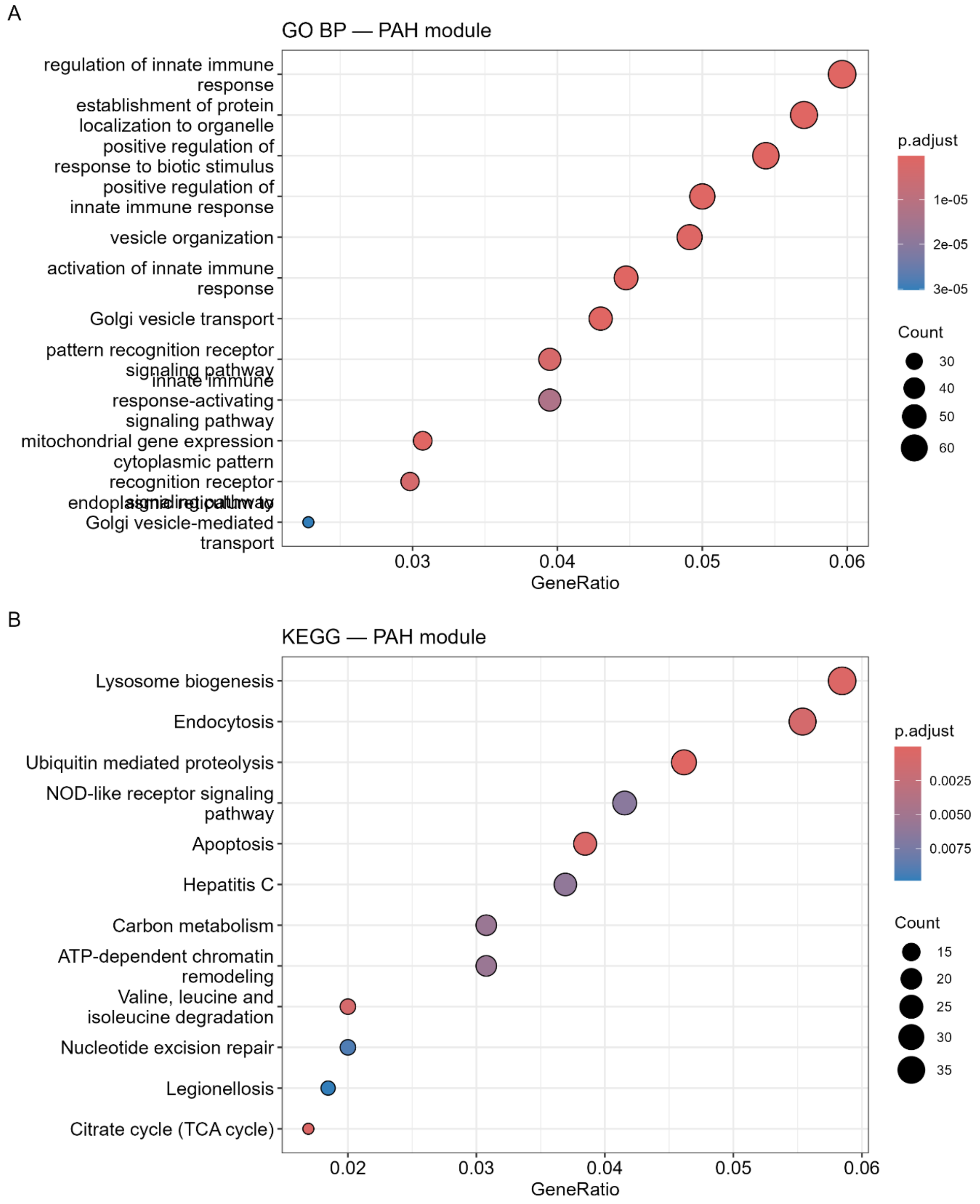

Figure S2. Functional enrichment of the PAH co-expression module. (A) Gene Ontology biological-process terms and (B) KEGG pathways enriched among genes of the ANKRD46-containing, diagnosis-associated module of GSE33463 (PAH) (clusterProfiler, Benjamini–Hochberg adjusted $p < 0.05$); top terms ranked by adjusted p, dot size proportional to gene count. Immune terms annotated by KEGG under infectious-disease names reflect shared innate-immune and interferon-stimulated genes, not active infection. The corresponding BD module returned no GO biological-process terms and only a single KEGG and a single Reactome term, and is therefore not shown.
